# Black Esophagus Under the Scope: Gurvits Syndrome in a Patient With Diabetic Ketoacidosis

**DOI:** 10.7759/cureus.81745

**Published:** 2025-04-05

**Authors:** Nayef Alkhalil, Ali R Chaitou, Abdullah Al Omary

**Affiliations:** 1 Division of Gastroenterology and Hepatology, Lebanese University Faculty of Medical Sciences, Beirut, LBN; 2 Division of Gastroenterology and Hepatology, American University of Beirut Medical Center, Beirut, LBN; 3 Division of Gastroenterology and Hepatology, Rafik Hariri University Hospital, Beirut, LBN

**Keywords:** acute esophageal necrosis (aen), acute necrotizing esophagitis, black esophagus, diabetic ketoacidosis (dka), esophageal ulcer, gurvits syndrome, melena, uncontrolled diabetes mellitus, upper gastrointestinal bleeding

## Abstract

A 60-year-old woman with a history of uncontrolled type 2 diabetes mellitus (DM), hypertension, and coronary artery disease was admitted to the intensive care unit due to diabetic ketoacidosis (DKA) triggered by urosepsis. During her stay, she developed upper gastrointestinal bleeding, manifesting as melena. Esophagogastroduodenoscopy revealed circumferential necrosis of the mid-to-distal esophagus, consistent with acute esophageal necrosis (AEN). The patient received supportive treatment, including proton pump inhibitor therapy and sucralfate. A follow-up gastroscopy four weeks later showed a resolution of necrosis but persistent mild esophagitis. This case highlights AEN as a rare complication in patients with uncontrolled type 2 DM and DKA.

## Introduction

Acute esophageal necrosis (AEN), also called "Gurvits syndrome," "black esophagus," or "acute necrotizing esophagitis," is a rare disorder characterized by ischemia and necrosis of the esophageal mucosa. It was first described in the medical literature in 1990 [1]. However, it has gained recognition due to the work of Gurvits et al. in 2007, providing a structured approach to identify AEN's risk factors, diagnosis, and pathogenesis [2]. AEN can be fatal; however, its incidence between 1993 and 2011 reached only 0.28% [3]. Notably, the disease disproportionately affects males more and typically presents in the sixth decade of life, with an average age of onset of around 67 years [4]. Its exact cause remains unknown but probably involves a combination of factors, including mucosal ischemia, exposure to gastric acid, and a weakened mucosal barrier in critically ill patients [4,5].

AEN typically presents with upper gastrointestinal bleeding, with hematemesis and melena being the most common symptoms [4,5]. Mortality rates associated with AEN vary, with some studies reporting figures as high as 32%, likely due to underlying comorbidities [4,5]. However, more recent data suggests a mortality rate closer to 6%, primarily related to esophageal perforation [2,4,5]. While a standardized treatment regimen for AEN is lacking, current approaches focus on managing coexisting conditions, fluid resuscitation, blood sugar control, administration of proton pump inhibitors, and parenteral nutrition [4,5].

DKA is reported to be linked to AEN in the current literature. A total of 31 case reports of AEN in the setting of DKA were reported from 2014 to 2022 [6]. The exact mechanism remains unclear, but several theories have been proposed. Patients with long-standing diabetes mellitus (DM), regardless of its type, are at increased risk of atherosclerosis, leading to compromised blood flow and ischemia [4,5]. Additionally, malnutrition, hemodynamic instability, and hyperglycemia associated with DKA can contribute to low blood flow and a weakened mucosal barrier, making the esophagus more susceptible to injury from stomach acid [4,5,7]. Furthermore, DKA can cause stagnation of gastric fluids, leading to increased gastroesophageal reflux and accelerating esophageal necrosis, in addition to fluid loss characteristic of DKA, which can potentially lead to hypoperfusion of the lower esophageal segments due to its relatively poor vascularization from the gastric artery branches in comparison to the proximal and middle segments [4,5].

This article reports the case of a patient with AEN that developed as a complication of DKA in the context of poorly controlled type 2 DM and urosepsis. The patient received successful treatment, preventing the progression to permanent esophageal damage.

## Case presentation

A 60-year-old woman with a history of long-standing uncontrolled type 2 DM, hypertension, and coronary artery disease presented to the emergency department (ED) with generalized fatigue, chills, dysuria, and bilateral flank pain. Her condition deteriorated while in the ED as she developed a decreased level of consciousness and got intubated, and a nasogastric tube (NGT) was inserted. Her blood pressure reached 90/70 mmHg in the ED but did not drop further, and aggressive intravenous (IV) fluid resuscitation was initiated. The glucometer in the ED detected "HIGH" (i.e., > 600 mg/dL), and the laboratory workup revealed severe metabolic acidosis (HCO_3_^-^ = 10 mEq/L), the presence of ketone bodies in the blood, and pyuria on urinalysis, prompting admission to the intensive care unit for the management and treatment of DKA precipitated by pyelonephritis and urosepsis (Table 1). The patient's antidiabetic therapy consisted of just metformin 500 mg orally twice daily.

**Table 1:** The patient's laboratory test results upon her presentation to the emergency department.

Lab Test	Result	Reference Range
White blood cell (WBC) count	20,000	4,500-11,000/mm^3^
Hemoglobin	12.6	12.5-16.5 g/dL
Platelet count	220,000	150,000-450,000/mm^3^
Creatinine	1.99	0.6-1.2 mg/dL
Blood urea nitrogen (BUN)	86	7-20 mg/dL
Sodium	145	136-146 mEq/L
Potassium	3.91	3.5-5 mEq/L
Chloride	101	95-105 mEq/L
Bicarbonate	10	22-28 mEq/L
C-reactive protein (CRP)	208.6	8-10 mg/L
Alanine transaminase (ALT)	22	10-33 U/L
Aspartate aminotransferase (AST)	20	10-33 U/L
Bilirubin total	0.26	0.1-1 mg/dL
Partial thromboplastin time (PTT)	28.8	25-35 seconds
International normalized ratio (INR)	1.04	0.8-1.1
HbA1c	11.4	≤ 5.7%
pH	7.12	7.4

On day two of her admission, the patient developed coffee-ground emesis via the NGT and black tarry stools. The gastroenterology team was consulted. The physical examination revealed a conscious but disoriented and pale patient with a soft and non-tender abdomen. A digital rectal examination confirmed the presence of melena.

**Figure 1:**
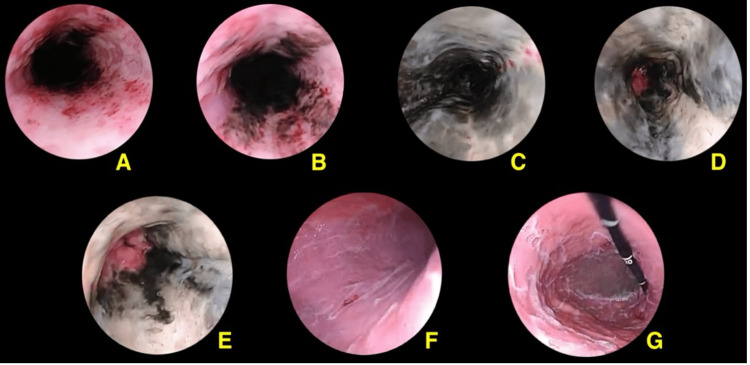
Esophagogastroduodenoscopy snapshots. (A,B) middle esophagus; (C,D) distal esophagus; (E) gastroesophageal junction; (F) stomach direct vision; (G) stomach retro-vision.

Esophagogastroduodenoscopy (EGD) was performed, demonstrating circumferential necrosis of the mid-distal esophagus with linear ulcerations sparing the gastroesophageal junction (GEJ) (Figures 1A-E). Erosive gastroduodenitis with abundant nonspecific white matter was observed in the stomach (Figures 1F, 1G), with no gastric or esophageal varices. Biopsies were limited to the stomach and duodenum due to concern for esophageal perforation. Subsequent pathology results showed normal gastric and duodenal mucosa.

The patient's clinical presentation and EGD findings were consistent with AEN. Therefore, a treatment regimen focused on supportive care was implemented. This included IV fluids for hydration, electrolyte correction, and insulin therapy for DKA management. To allow for esophageal healing, she was maintained on nothing by mouth (NPO) for three days, followed by a gradual diet advancement as tolerated. Using an electronic infusion pump, IV omeprazole 8 mg/hour was continuously administered for the initial 72 hours and was transitioned afterward to split IV dosing of 80 mg twice daily along with oral sucralfate for continued management. Additionally, IV fluconazole was administered empirically for two weeks to provide antifungal prophylaxis against potential fungal esophagitis. A control gastroscopy four weeks later showed complete resolution of the prior necrosis but revealed mild residual lower-third esophagitis with ulcers and white exudates.

## Discussion

The diagnosis of AEN relies primarily on endoscopic findings, specifically the circumferential black discoloration of the esophageal mucosa with a sharp demarcation at the GEJ [1,2,4,5,7]. Studies suggest that nearly 90% of AEN patients have uncontrolled hyperglycemia [7]. Other associated conditions include vascular disorders, hypertension, chronic kidney disease, cancer, malnutrition, and gastric outlet obstruction. The most common clinical presentation is upper gastrointestinal bleeding, with most patients experiencing hematemesis, coffee-ground emesis, and/or melena, similar to our case. Other commonly reported gastrointestinal symptoms include epigastric pain, chest pain, nausea, vomiting, and swallowing difficulties. Less frequent symptoms include fever, hypotension, and decreased level of consciousness. Laboratory findings are often non-specific, but abnormalities like anemia, leukocytosis, and high blood lactate levels have been reported [4,5].

Our patient was on a metformin regimen only as a treatment for her type 2 DM, which reflects an undertreatment regarding her elevated HbA1c level. She did not require the use of vasopressors, her blood pressure did not drop below 90/70 mmHg since her presentation, and she was stabilized by aggressive IV hydration only, which excludes the factor of systemic hypoperfusion in this case. Further, the NGT insertion was smooth without any complication reported, which excludes the probability of direct mucosal injury of the esophagus due to its insertion. The status of DKA with its hemodynamic complications remains the only flagrant culprit for the trigger of AEN in our case.

There is no established standard of care for AEN. Management focuses on addressing the underlying medical condition, IV hydration, total parenteral nutrition (TPN), and protecting the esophagus from further damage with high-dose IV proton pump inhibitors. Enteral feeding should be avoided due to the high risk of esophageal perforation. Similarly, prophylactic antibiotics are not indicated unless the patient shows signs of a serious and active infection. IV fluconazole was administered prophylactically for the prevention of esophageal candidiasis in this case of immunosuppression due to poorly controlled DM and mucosal necrosis, despite not being a standard regimen in such cases. The most common long-term complication of AEN is esophageal stricture, affecting approximately 10% of patients [4,5]. Fortunately, a follow-up endoscopy for our patient did not reveal any stricture formation after four weeks.

## Conclusions

Clinicians managing DKA, especially in older adults with poorly controlled diabetes or significant comorbidities, should consider AEN as a possible cause of upper gastrointestinal bleeding. Timely diagnosis and prompt treatment are essential for achieving the best outcomes and preventing complications.
